# In Memoriam: Sydney Finegold (1921-2018)

**DOI:** 10.1080/16512235.2018.1533367

**Published:** 2018-10-23

We express our sincere condolences for Professor Sydney Finegold.

Professor Finegold was a father in education for all of us. He was the first elected President and founder of our Society, called SIMED in the years 1970’s.

He was the one who proposed SOMED as the new last name of our Society at the minutes of the 1988th, Porto Conte, Sassari, Italy Meeting of our Society .He felt that a change should be made to accommodate areas of microbial ecology in addition to intestinal ecology.

Eminent Professor at UCLA, Medical School, he was the leader of the Anaerobic Microbiology, as well as founder of the Anaerobes Society of The Americas(ASA).

The title ”Father of Anaerobes” cannot yield the greatness of his offers to the scientific community with leading publications in the domain.

His most important contribution to society was the establishment of connections between scientific research all over the world, as tools for strengthening cohesion in the area of Anaerobic Microbiology. Moreover, he was the main driving force with regard to the writing of important Infectious Diseases and Anaerobic Identification Manuals.

Professor Finegold always wholeheartedly offered his knowledge to those who needed it. He believed in approaching scientific and social issues in an interdisciplinary manner. He was always open to cooperation and greatly inspired teamwork and of help to different scientists all over the world.

Professor’s Finegold research interests are relevant all around the Scientific World, and everyone hopes that there will be a multinational application of his work in the years ahead. He was a very kind, unpretentious and genuine person.

I had the opportunity to work with him and appreciated from near its enormous burden of knowledge in the field, as well as his human face and kindness.

Professor Finegold was a great personality which enlightened our scientific area. Moreover, he showed everyone that the job of a teacher is not a job but public service.

Our scientific community lost his Chief- leader.

Sincere condolences to his people, wife, children and the whole family.

Eugenia Bezirtzoglou (on behalf of the SOMED)

Professor in Microbiology, MD, PhD

President SOMED

